# Preflight Calibration and Performance Assessment of the Geostationary Interferometric Infrared Sounder (GIIRS) Onboard the FengYun-4B Satellite

**DOI:** 10.3390/s26092763

**Published:** 2026-04-29

**Authors:** Lu Lee, Libing Li, Yaopu Zou, Zhanhu Wang, Changpei Han, Liguo Zhang, Lei Ding

**Affiliations:** 1 Innovation Center for FengYun Meteorological Satellite, Key Laboratory of Radiometric Calibration and Validation for Environmental Satellites, National Satellite Meteorological Center, China Meteorological Administration, Beijing 100081, China; 2 Key Laboratory of Infrared System Detection and Imaging Technology, Shanghai Institute of Technical Physics, Chinese Academy of Sciences, Shanghai 200083, China; lilibing@mail.sitp.ac.cn (L.L.); zyp@mail.sitp.ac.cn (Y.Z.); wangzhanhu@mail.sitp.ac.cn (Z.W.); changpei_han@mail.sitp.ac.cn (C.H.); leiding@mail.sitp.ac.cn (L.D.); 3 Shanghai Institute of Satellite Engineering, Shanghai 200240, China; zlg_1987@buaa.edu.cn

**Keywords:** spectrometer, weather satellite, infrared sensor, calibration, sensitivity, correction

## Abstract

**Highlights:**

**What are the main findings?**

- Preflight tests verified that the NEdR, spectral calibration, and radiometric calibration accuracies of the FY-4B/GIIRS fulfill all design specifications, and the effective dynamic range for observations was estimated via preflight temperature-variable blackbody tests.
- The evaluation of nonlinear response in LWIR and MWIR bands resulted in a specialized correction method for the LWIR detector, which successfully reduced the radiometric calibration error from >1 K to approximately 0.2 K.

**What are the implications of the main findings?**

- The effective observation dynamic range and the baseline performance characterized through preflight calibration allow the Level-1 data users to understand the instrument’s capabilities and ensure high-quality data for meteorological applications.
- The implementation and verification of the LWIR nonlinearity correction provide a critical methodology for mitigating detector-induced biases, significantly enhancing the precision of atmospheric temperature vertical structure retrievals.

**Abstract:**

The Geostationary Interferometric Infrared Sounder (GIIRS) onboard the FengYun-4B weather satellite provides critical upwelling atmospheric infrared radiance. To address the limitations of the previous sounder (FY-4A/GIIRS) in terms of spatial resolution and spectral coverage, FY-4B/GIIRS has increased the spatial resolution to 12 km and added more spectral channels in the long-wave band to enhance the observation details and information content of weather systems. To evaluate its baseline performance, a comprehensive preflight test campaign—encompassing spectral and radiometric assessments—was conducted in a thermal vacuum (TVAC) chamber. Spectral characterization via laser measurements confirmed the instrument spectral response function (ISRF) is highly consistent with the theoretical cardinal sine function (sinc). Gas-cell tests demonstrated that, after correcting for off-axis effect, the spectral calibration errors are on average less than 5 ppm, validated against Line-By-Line Radiative Transfer Model (LBLRTM) simulations. The radiometric calibration employed temperature-variable blackbodies for noise performance and radiometric accuracy assessments. The radiometric sensitivity, characterized by Noise Equivalent differential Radiance (NEdR), is less than 0.5 and 0.1 mW/(m^2^·sr·cm^−1^) in the long-wave infrared (LWIR) and mid-wave infrared (MWIR) bands, respectively. To address the LWIR detector nonlinearity, an iterative polynomial fitting algorithm based on spectral responsivity invariance was implemented. This correction reduces the radiometric deviation from >1.0 K to ~0.2 K, meeting the 0.7 K accuracy requirement across a 180–315 K dynamic range. Conversely, the MWIR band exhibits high linearity but is limited by noise when observing low-temperature scenarios and can only meet the 0.7 K requirement within the range of 250 to 315 K.

## 1. Introduction

The Geostationary Interferometric Infrared Sounder (GIIRS) onboard the FengYun-4B (FY-4B) weather satellite is a hyperspectral infrared (IR) sounder utilizing a Fourier transform spectrometer to measure upwelling atmospheric infrared radiance at a spectral sampling interval of 0.625 cm^−1^. These data are instrumental for atmospheric temperature and moisture retrieval, Numerical Weather Prediction (NWP) data assimilation, and also trace gas monitoring. High-accuracy hyperspectral radiance measurements are the cornerstone of modern NWP and atmospheric chemistry studies. For NWP data assimilation, even sub-Kelvin systematic biases in radiance can lead to significant errors in the retrieved temperature and humidity profiles, ultimately degrading the forecast skill. Similarly, the precise retrieval of trace gas concentrations (e.g., NH_3_, CO, and CH_4_) requires exceptional spectral stability and radiometric fidelity to distinguish faint molecular absorption features from the background thermal emission and instrumental noise. Consequently, a rigorous preflight calibration is not merely a technical requirement but a prerequisite for ensuring the traceability and consistency of Level-1 products, which directly determines the effectiveness of downstream meteorological and environmental applications. Therefore, it is critical to characterize instrument performance and establish and refine calibration algorithms through rigorous pre- and post-launch calibration and validation (Cal/Val) activities.

The preflight calibration of hyperspectral infrared sounders relies on well-established, rigorous methodologies developed over decades. Foundational Low Earth Orbit (LEO) instruments, such as AIRS, IASI, and CrIS, have established the industry baseline for radiometric and spectral calibration, successfully utilizing techniques such as laser-based instrument spectral response function (ISRF) characterization and blackbody-based radiometric calibration. While the fundamental physical principles of these calibration approaches are universally applied, their specific implementation must be rigorously tailored to address the universal hardware challenges of each specific payload, such as off-axis frequency shifts in large focal plane arrays and the nonlinear response inherent to HgCdTe detectors. Rather than proposing a novel theoretical calibration paradigm, this study details the comprehensive application of these proven, high-fidelity calibration methodologies to the FY-4B/GIIRS thermal vacuum (TVAC) campaign. The rationale is to ensure that the instrument’s baseline performance is strictly verified against these gold-standard physical principles, thereby meeting the stringent accuracy requirements for geostationary orbit before launch.

The primary objective of the preflight calibration campaign is to establish a high-fidelity baseline for the FY-4B/GIIRS (or GIIRS-B) through traceability to primary physical standards. First, we hypothesize that the accuracy spectral response characterized by the ISRF can be measured utilizing monochromatic lasers traceable to national metrology standards. Second, for spectral frequency positioning, the known absorption lines of target gases are employed as prior truths to evaluate the frequency shifts caused by off-axis effects of the focal plane array. By correcting the off-axis scaling factor (the cosine of the off-axis angle), the measured spectra can be accurately mapped to a standard frequency grid. Finally, regarding radiometric performance, a temperature-variable blackbody with precisely calibrated emissivity is utilized as a simulator of Earth scenes. Based on the Planck equation, the blackbody radiance provides a reference to quantify and verify the radiometric accuracy—including the characterization of detector nonlinearity and estimation of the dynamic range. These systematic Cal/Val efforts establish a baseline for instrument characterization and performance assessment, ensuring that the GIIRS-B instrument performance fulfills the stringent spectral and radiometric specifications required for advanced atmospheric sounding, and provide the initial calibration coefficients for on-orbit instrument calibration and data processing.

This paper is organized as follows. Section 2 gives an overview of the GIIRS-B instrument and its measurement requirements. Section 3 describes the layout of the GIIRS inside the thermal vacuum (TVAC) chamber and the setup and methods for spectral and radiometric calibrations. Section 4 addresses the calibration results and the instrument performance. Finally, Section 5 summarizes the results with an emphasis on lessons learned.

## 2. Instrument and Measurements

**Figure 1:**
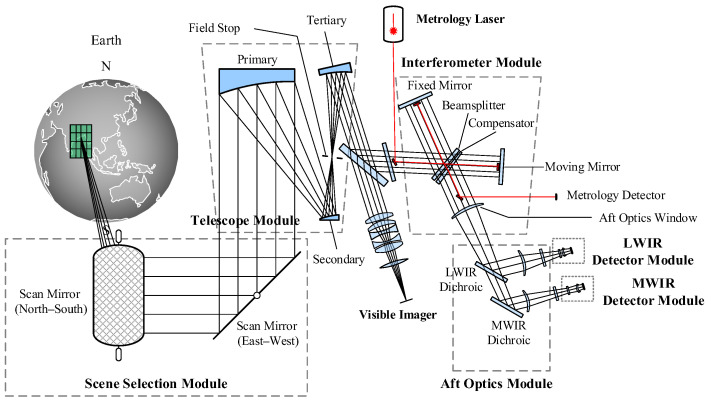
Optical schematic of FY-4B/GIIRS.

**Figure 2:**
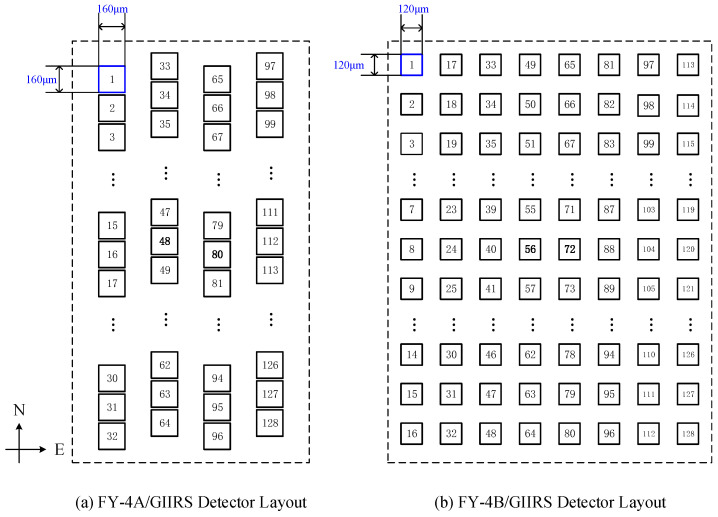
Layout comparison of GIIRS-A and GIIRS-B infrared detectors.

The FY-4B/GIIRS was designed by the Shanghai Institute of Technical Physics of the Chinese Academy of Sciences (SITP and CAS). Unlike its predecessor, the FY-4A/GIIRS (GIIRS-A), which served primarily as a scientific demonstration, GIIRS-B is designed for operational service by the China Meteorological Administration [1]. As shown in Figure 1, the instrument architecture comprises a two-mirror orthogonal scanner, a telescope, a Michelson interferometer, aft optics, two infrared detector arrays, and a visible-light panchromatic imager. The panchromatic imager co-shares the two-mirror scanner and the telescope optics to facilitate navigation and cloud detection. As illustrated in Figure 2b, the GIIRS-B detector employs a sparse 16 × 8 array matrix. Compared to the 32 × 4 array matrix used in GIIRS-A (shown in Figure 2a), the pixel size is reduced from 160 μm to 120 μm, enhancing the nadir spatial resolution from 16 km to 12 km (each pixel corresponds to a single instantaneous field of view, hereinafter referred to as FOV). A one-pixel gap between adjacent elements is incorporated to minimize optical crosstalk. The equivalent coverage area of the detector array contour at the nadir field of regard (FOR) is about 384 × 192 km. The IR detectors are cooled and maintained at an operational temperature of 65 K by a Sterling cooler, while the aft optics are cooled to approximately 200 K by a radiative cooler to suppress the thermal self-emission caused by the optical elements between interferometer and IR detectors.

According to the research on atmospheric infrared sounding technology in geostationary (Geo) orbit, it is found that a Geo sounder achieves the temperature and water vapor information more efficiently using two, rather than three, distinct spectral bands [2]. This selection of a two-band configuration represents a trade-off between atmospheric sounding efficiency and geostationary platform resource constraints. Historically, polar-orbiting (Leo) sounders like IASI [3], CrIS [4], and HIRAS [5] utilized a three-band approach to provide continuous spectral coverage. However, the primary mission of the Geo sounder is the high-temporal-resolution retrieval of atmospheric temperature and moisture vertical structures. For temperature profiling, the LWIR band effectively captures the CO_2_ and O_3_ absorption features. For moisture profiling, the selected MWIR range (covering the 4.6–6.25 μm water vapor absorption band) offers a significantly cleaner spectral window compared to the 6.25–8.5 μm range (a mirrored water vapor absorption band), the latter being complicated by the absorption lines of trace gases such as CH_4_, SO_2_, and N_2_O. From an engineering perspective, Geo sounders transition from the small-scale detector arrays used in LEO (typically 4 to 9 pixels) to large-scale focal plane arrays (e.g., the 16 × 8 matrix in GIIRS-B) to meet spatial coverage requirements. The data throughput, power consumption, and thermal cooling capacity required for three bands would impose excessive demands on the satellite bus. Consequently, the two-band design of GIIRS facilitates the core sounding mission while maintaining a sustainable balance of weight, power, and data downlink volume for long-term geostationary operations.

**Figure 3:**
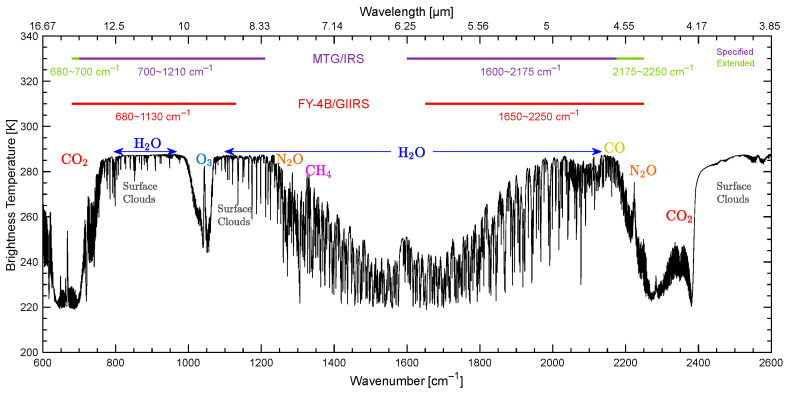
Spectral coverage of geostationary infrared sounders.

Therefore, the FY-4/GIIRS uses two IR detector arrays: one covers the long-wave IR band (LWIR) from 680 to 1130 cm^−1^ and the other covers the mid-wave band (MWIR) from 1650 to 2250 cm^−1^ (shown in Figure 3). Compared with the LWIR band (700~1130 cm^−1^) of GIIRS-A, GIIRS-B extends 20 cm^−1^ towards the long-wave side, which is beneficial for measuring upper atmosphere temperature. Table 1 lists the primary requirements of GIIRS-B.

## 3. Preflight TVAC Test Setup and Method

### 3.1. Test Setup in TVAC Chamber

**Figure 4:**
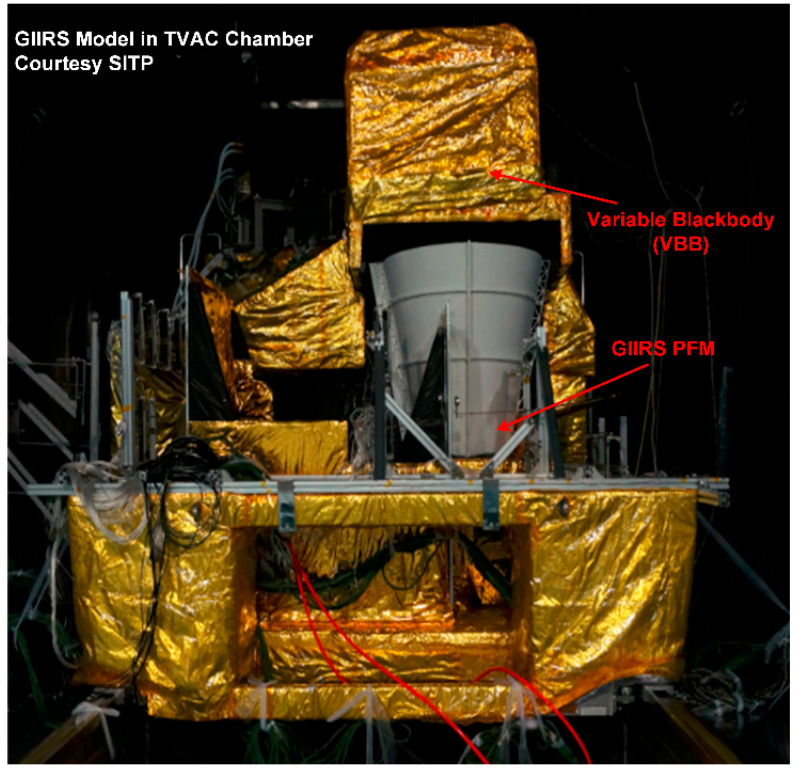
FY-4B/GIIRS proto flight model (PFM) in the TVAC chamber.

**Figure 5:**
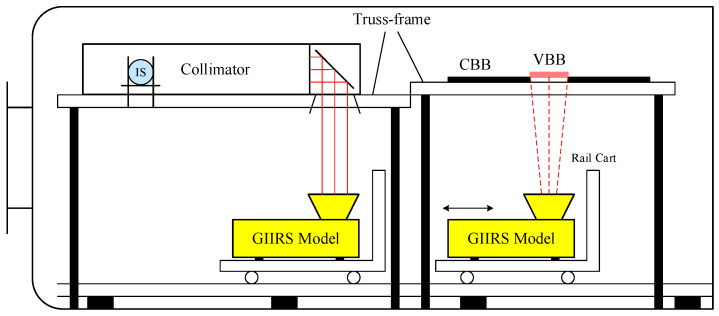
Schematic of the calibration test setup inside the TVAC chamber.

The preflight calibration measurements for GIIRS-B were conducted in a 4 m diameter TVAC chamber at the Shanghai Institute of Satellite Engineering (SISE). The TVAC test setup is presented in Figure 4; the instrument and a carrier trolley (a rail cart) were installed inside the chamber, and the instrument was configured with its telescope aperture viewing zenith-ward through the two-mirror scanner. The calibration sources for spectral and radiometric tests were mounted on an internal truss-frame, and transitions between spectral and radiometric test configurations were switched by translating the trolley carrying the instrument along the guide rail. The schematic of the test setup inside the TVAC chamber is presented in Figure 5.

### 3.2. Spectral Calibration Test Setup and Method

The GIIRS-B radiometric accuracy depends on accurate spectral calibration, including instrument spectral response function (ISRF) determination and frequency correction of the wavenumber grid. The primary design feature of the GIIRS related to spectral calibration is the geometry layout of the detector array. The interferometer axis is nominally perpendicular to the center of the detector array, and pixels detect the off-axis radiation and produce the so-called off-axis interferograms with shorter optical path differences (OPDs) than the nominal on-axis OPD. The off-axis angle varies with the distance of the pixel deviating away from the optical axis and is generally removed by the software correction. The calibrated radiances for all pixels on a detector array of each band are on a common wavenumber grid that has the same ISRF profile described by a cardinal sine function (with the boxcar apodization).

The off-axis corrections and frequency calibration depend on accurate knowledge of the GIIRS detector array geometry and the wavelength of the metrology laser used for interferogram sampling. The metrology laser with a nominal wavelength of 852.356 nm is precisely frequency-stabilized and temperature-controlled. Although the metrology laser is precisely frequency-stabilized, any slight optical misalignment between the laser path and the infrared beam alters the effective sampling interval, causing a uniform systematic frequency shift across all pixels. An “equivalent wavelength” mathematically compensates for this bulk shift. This equivalent wavelength is derived by measuring the relative spectral shift of known gas absorption lines during the TVAC gas-cell test and scaling the nominal laser wavelength accordingly. If necessary, this equivalent wavelength will be further tuned according to the atmospheric fingerprint spectrum during the post-launch Cal/Val activities [6].

**Figure 6:**
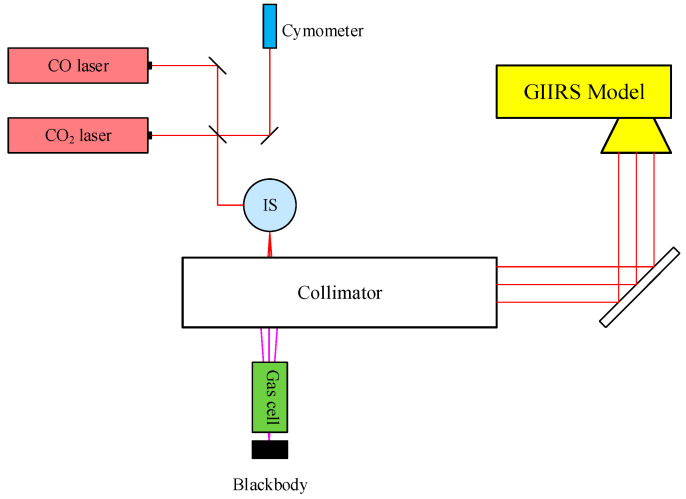
Schematic of the test setup for spectral calibration.

During the TVAC testing for spectral calibration, the ISRF knowledge was characterized and validated by a quasi-monochromatic laser source for each band, while the off-axis parameters associated with the detector array geometry and the laser equivalent wavelength were determined by gas-cell measurements. It should be noted that the detector geometry was characterized by the pixel FOV angles. The test was performed in advance outside the TVAC chamber and in a room-temperature ambience by scanning the detector’s field of view with an illuminated slit. The initial off-axis parameters were determined by the cosine values of these FOV angles. Physically, the OPD of an off-axis pixel is scaled by the cosine of its deviation angle relative to the on-axis OPD. Therefore, applying these specific cosine values is strictly required to accurately rescale the individual wavenumber grid for each off-axis detector pixel. The test setup, as shown in Figure 6, consisted of two quasi-monochromatic lasers sharing a metrology calibrated cymometer, one Integrating Sphere (IS), two separate gas cells filled with ammonia and carbon monoxide respectively, a background blackbody with variable temperature, and a collimator. The 10.6 μm CO_2_ laser was used to measure the LWIR ISRF shape, while the 5.4 μm CO laser was used to measure the MWIR ISRF shape. Although the laser tests and the gas-cell tests shared the same collimator, they were performed independently.

In order to determine the accurate detector array geometry and further derive the off-axis correction coefficients, the transmittance spectra of NH_3_ and CO, which have spectral features of absorption lines in the LWIR and MWIR bands, respectively, were recorded. The measurement of gas transmittance is based on the Beer–Lambert law and the instrument radiometric response equation. The transmittance of a single gas is defined as

(1) $$\tau_{gas}\left(\sigma \right)=\frac{L\left(\sigma \right)}{L_{0}\left(\sigma \right)}=exp\left(-k\left(T,p,\sigma \right)\cdot N\cdot L\right).$$

where *τ*_gas_ is the transmittance (dimensionless), *σ* is the wavenumber (cm^−1^), and *L*_0_ and *L* are the incident and transmitted radiation intensities (W · m^−2^ · sr^−1^ · (cm^−1^)^−1^), respectively. *k* (cm^2^ · mole^−1^) is the effective absorption area (molecular absorption cross-section) of gas molecules for radiation at wavenumber *σ* under the conditions of temperature *T* and pressure *p*. It incorporates both the spectral line intensity and line shape functions (such as Lorentz, Doppler, and Voigt line shapes). *N* is the number density of molecules (mole · cm^−3^). *L* is the effective optical path (cm), that is, the distance between the two windows of the gas cell.

In the spectral calibration test, the gas transmittance is derived by observing the gas cell under four states. First, take a cold blackbody (with a temperature of 295 K) as the radiation source; the instrument observes the radiance that passes through the empty gas cell (the cell has been evacuated to a vacuum state). The raw spectral signal measured by the instrument (unit: digital count) is denoted as *C*_ETC_. Second, fill the gas cell with a single gas (such as NH_3_) and record the instrument measurement spectrum as *C*_FTC_. Then, replace the cold blackbody with a hot blackbody (with a temperature of 310 K) and measure the empty gas spectrum *C*_ETH_ and the gas-filled spectrum *C*_FTH_ again, respectively. The four measured raw spectra, *C*_ETC_, *C*_FTC_, *C*_ETH_, and *C*_FTH_, can be expressed as follows:

(2) $$\begin{array}{c} \begin{array}{l} C_{ETC}\left(\sigma \right)=R_{ins}\left(\sigma \right)\cdot L_{BB}^{C}\left(\sigma \right)+C_{offset}\left(\sigma \right)\\ C_{FTC}\left(\sigma \right)=R_{ins}\left(\sigma \right)\cdot \left[{\tau_{gas}\left(\sigma \right)\cdot L}_{BB}^{C}\left(\sigma \right)+\left(1-\tau_{gas}\left(\sigma \right)\right)\cdot L_{gas}\left(\sigma \right)\right]+C_{offset}\left(\sigma \right) \end{array}\\ \begin{array}{l} C_{ETH}\left(\sigma \right)=R_{ins}\left(\sigma \right)\cdot L_{BB}^{H}\left(\sigma \right)+C_{offset}\left(\sigma \right)\\ C_{FTH}\left(\sigma \right)=R_{ins}\left(\sigma \right)\cdot \left[{\tau_{gas}\left(\sigma \right)\cdot L}_{BB}^{H}\left(\sigma \right)+\left(1-\tau_{gas}\left(\sigma \right)\right)\cdot L_{gas}\left(\sigma \right)\right]+C_{offset}\left(\sigma \right) \end{array} \end{array}.$$

where *R*_ins_ is the instrument spectral responsivity, *C*_offset_ is the electronic offset spectrum (unit: digital count), and $L_{BB}^{C}$ and $L_{BB}^{H}$ are the background radiance spectra of the cold and hot blackbodies, respectively. *L*_gas_ is the gas self-emission with its emissivity being (1 − *τ*_gas_). It should be noted that, for simplicity, the radiation of the gas-cell window was already included in the blackbody background radiance. Furthermore, as the length of the gas cell was only 20 cm, and the gas concentration was relatively low, the gas layer was simplified to a single layer and the complex multi-layer gas radiation model was no longer employed. As can be seen from the equations of *C*_FTC_ and *C*_FTH_, when the gas cell is filled with gas, the effective radiation received by the instrument comes partly from the transmitted radiance of the background blackbody and partly from the gas self-emission. From the above equations, the gas transmittance can be derived as

(3) $$\tau_{gas}\left(\sigma \right)=\frac{C_{FTH}\left(\sigma \right)-C_{FTC}\left(\sigma \right)}{C_{ETH}\left(\sigma \right)-C_{ETC}\left(\sigma \right)}.$$

To evaluate the accuracy of the measured spectrum and derive the off-axis correction coefficients, the simulated transmittance computed by the Line-By-Line Radiative Transfer Model (LBLRTM) is usually used as the reference truth value. The adoption of LBLRTM-simulated spectra as the truth reference for spectral calibration is due to its status as the “gold standard” of atmospheric radiative transfer. The LBLRTM strictly adheres to the Beer–Lambert law by performing line-by-line calculations on molecular absorption lines from the HITRAN database, enabling precise computation of gas transmittance and radiance. According to the Beer–Lambert law (Equation (1)), the input parameters of the LBLRTM include monochromatic gas absorption coefficients, gas-cell temperature, gas partial pressure, and optical path. It should be noted that the LBLRTM-simulated transmittances need to be converted into data with the same spectral resolution and wavenumber grid as GIIRS-measured data. That is, the simulated transmittances need to be convolved with the theoretical ISRF of GIIRS. Then, using the simulated transmittances as the reference truth, the Root-Mean-Square errors (RMS) of measured and computed transmittance are minimized by varying the laser wavelength and/or pixel off-axis parameters. When the RMS reaches its minimum, the equivalent laser wavelength and pixel off-axis parameter are determined and can be used for spectral off-axis correction.

### 3.3. Radiometric Calibration Test Setup and Method

**Figure 7:**
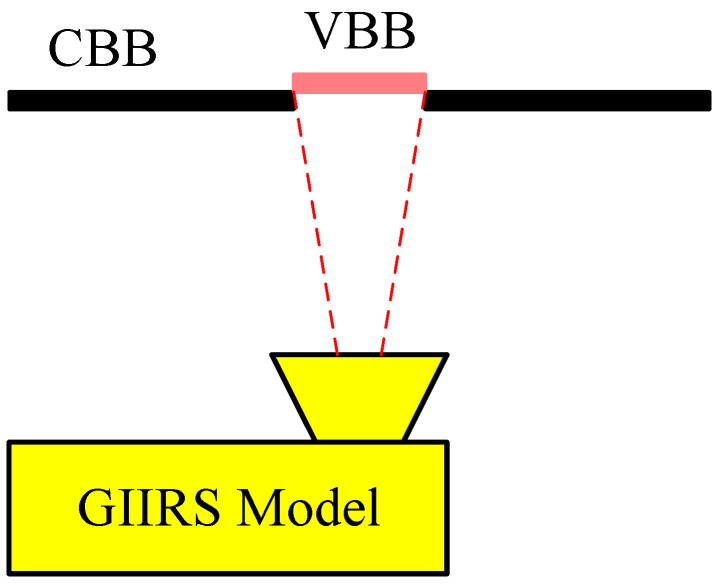
Schematic of the test setup for radiometric calibration.

Radiometric calibration is essentially a process of assigning exact physical radiance values [mW/(m^2^·sr·cm^−1^)] to the amplitudes [counts/cm^−1^] of the Fourier-transformed spectral data. Based on the experiences of radiometric calibration of some similar IR sounders (such as IASI, CrIS, and HIRAS), the FY-4B/GIIRS radiometric calibration also adopts the complex calibration method [7]. This complex calibration method is based on two-point characterization, which requires two calibration references with high and low temperatures, respectively. The knowledge of each calibration reference should be known or modelable radiance. In the practice of radiometric calibration, the instrument needs to frequently and periodically view the cold and the hot reference. In orbit, the cold reference is usually deep space (DS) and the hot is the onboard blackbody or the so-called Internal Calibration Target (ICT), while in preflight tests, to simulate the on-orbit environment, a cold blackbody (CBB) kept at around 80 K serves as the cold reference, and the ICT is still the hot reference. Additionally, a temperature-variable blackbody (VBB) is used to simulate the Earth scene with the view field being as same as the GIIRS views of the Earth in orbit (see Figure 7). The temperature of the VBB can vary from 180 K to 320 K at an interval of 10 K, which is sufficient to represent the dynamic range of the flight signals in terms of Top-Of-Atmosphere (TOA) radiances and instrument internal temperatures.

The radiometric calibration equation for the preflight test is expressed as follows [8]:

(4) $$L_{VBB}\left(\sigma \right)=\frac{C_{VBB}\left(\sigma \right)\;-\;\langle C_{CBB}\left(\sigma \right)\rangle }{C_{ICT}\left(\sigma \right)\;-\;\langle C_{CBB}\left(\sigma \right)\rangle }\cdot \left[L_{ICT}\left(T_{ICT},\sigma \right)-L_{CBB}\left(\sigma \right)\right]+L_{CBB}\left(\sigma \right),$$

where *L*_VBB_(*σ*) is the calibrated radiance of the VBB over the channel against wavenumber *σ*; *C*_VBB_(*σ*), *C*_ICT_(*σ*), and *C*_CBB_(*σ*) are the complex raw spectra by Fourier transform of the discrete sampled interferogram samples of the VBB, ICT, and CBB, respectively; *L*_ICT_ and *L*_CBB_ are the known radiance of ICT and CBB, which are calculated by the blackbody radiance model (based on Planck’s equation) at the known temperature of *T*_ICT_ and *T*_CBB_; and operator 〈·〉 denotes ensemble averaging over multiple consecutive spectrum samples to reduce random noise. The sample size is over 30. Since *C*_VBB_, *C*_ICT_, and *C*_CBB_ are complex values, the calibrated radiance *L*_VBB_ is also complex with its real part being the radiance spectrum required for atmospheric parameter retrieval, while the imaginary part is complete zero-mean system random noise. For a noise-free instrument, the imaginary part of the calibrated radiance is equal to zero for all channels. Although the imaginary part is discarded in the atmospheric applications, this property is very useful in radiometric calibration quality control to identify if the real part of the radiance is erroneous [4,9].

## 4. Results and Performance Assessment

### 4.1. Spectral Calibration

**Figure 8:**
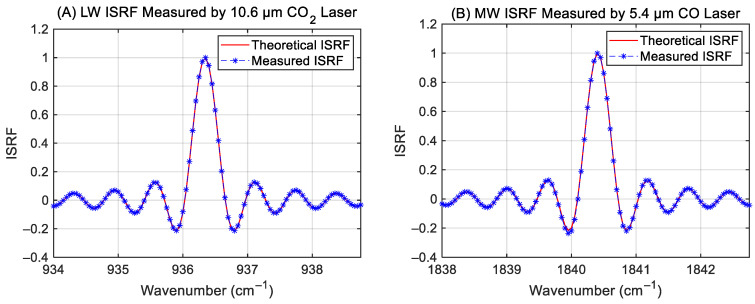
The ISRF shapes at (**A**) LWIR 10.6 μm, and (**B**) MWIR 5.4 μm, respectively. (Note: In order to reveal the continuous shape of the sinc function, the ISRF shapes are both interpolated sinc functions using the zero-padding technique of Fourier transform spectroscopy, and the corresponding spectral sampling interval is 0.1 cm^−1^.).

The measured ISRF was compared against the theoretical ISRF profile derived from the GIIRS-B estimation model. As shown in Figure 8, the ISRF shapes obtained via LWIR 10.6 μm CO_2_ laser and MWIR 5.4 μm CO laser measurements demonstrate excellent agreement with the theoretical sinc function.

**Figure 9:**
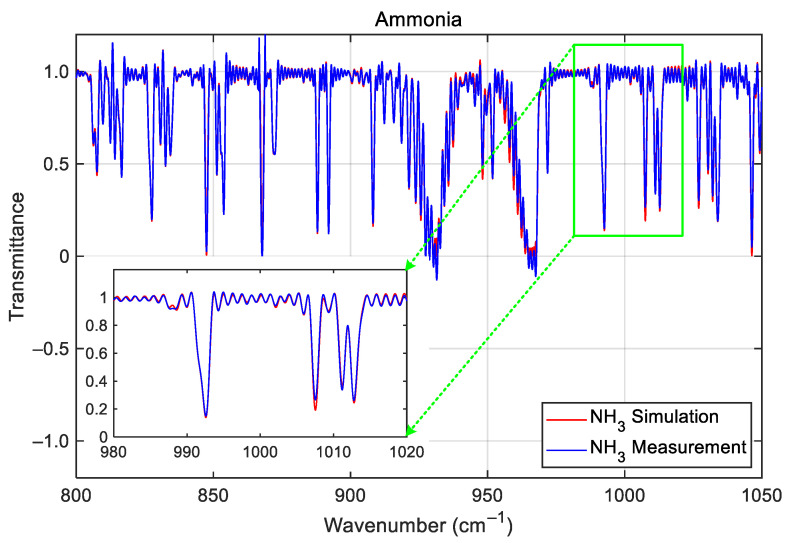
The calibrated gas-cell spectrum of NH_3_ taken with the GIIRS during TVAC and its comparison with the LBLRTM simulation. The zoomed inset shows the details of the absorption lines between 990 and 1020 cm^−1^.

**Figure 10:**
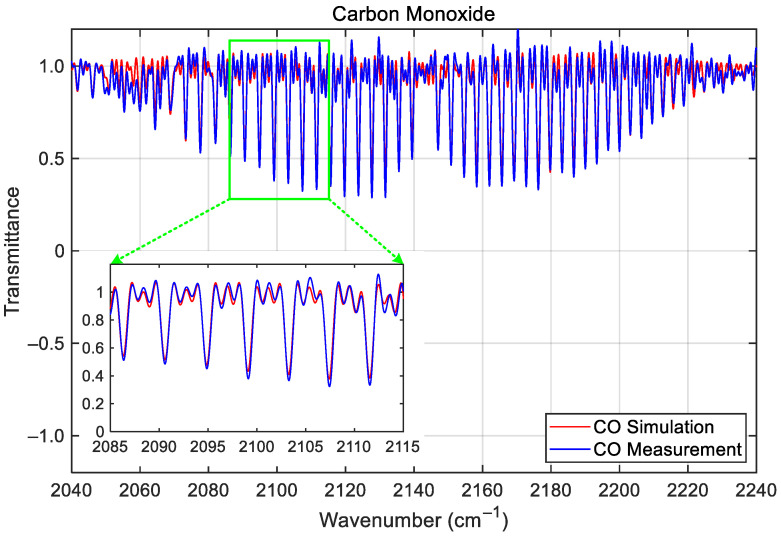
The calibrated gas-cell spectrum of CO taken with the GIIRS during TVAC and its comparison with the LBLRTM simulation. The zoomed inset shows the details of the absorption lines between 2085 and 2115 cm^−1^.

Following off-axis correction, the calibrated transmittance spectra for NH_3_ (LWIR) shown in Figure 9 and CO (MWIR) shown in Figure 10 aligned closely with LBLRTM simulations, respectively. The accuracy of the calibrated spectra is quantitatively evaluated by performing a spectral cross-correlation between the measured gas-cell spectra and the simulated transmittances given by the LBLRTM [6]. This cross-correlation process yields a correlation coefficient that serves as a metric for the quality of the spectral match; any systematic shift or distortion in the observed spectrum relative to the high-fidelity LBLRTM reference indicates a wavenumber calibration deviation *dσ* (cm^−1^). If necessary (for example, if the off-axis parameter measurements are very inaccurate), the preset off-axis parameters need to be iteratively adjusted to maximize the cross-correlation, thereby minimizing the remaining wavenumber deviations. Using this evaluation method, the resulting spectral deviations (the relative wavenumber error, *dσ*/*σ*_0_ × 10^6^; *σ*_0_ is the LBLRTM reference wavenumber) in the two bands of the GIIRS are on average below 5 ppm, well within the 10 ppm requirement.

### 4.2. Noise Performance

**Figure 11:**
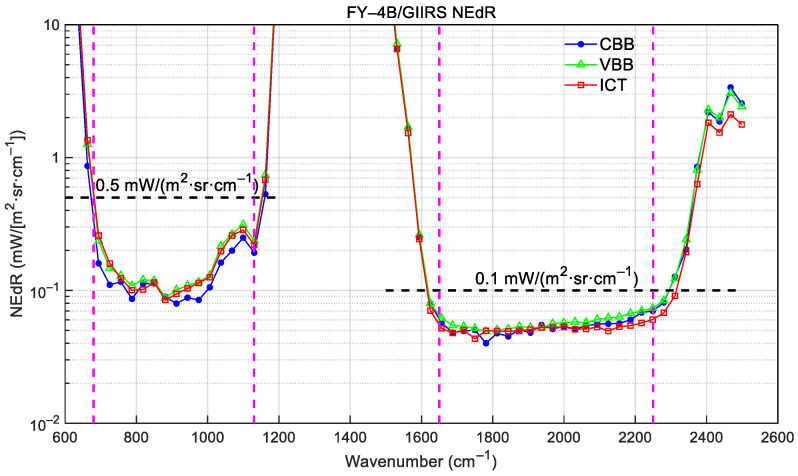
FY-4B/GIIRS NEdRs calculated from the calibrated radiances of CBB, VBB, and ICT. (The dashed lines define the nominal spectral ranges of the two bands with LWIR = 680~1130 cm^−1^ and MWIR = 1650~2250 cm^−1^).

The instrument sensitivity of the GIIRS is characterized by the Noise Equivalent differential Radiance (NEdR). The calculation equation for NEdR is expressed as follows [9]:

(5) $$NEdR\left(\sigma \right)=\sqrt{\frac{1}{M-1}\sum_{j=1}^{M}{\left[Re\left\{L_{j}\left(\sigma \right)\right\}-\langle Re\left\{L_{j}\left(\sigma \right)\right\}\rangle \right]}^{2}}.$$

where *j* is the sample number with a total amount of *M*, and Re{} denotes taking the real part of a complex item. The NEdR is calculated using not only the VBB calibrated radiance *L*_VBB_, but also the ones of CBB and ICT (*L*_CBB_ and *L*_ICT_). Figure 11 shows the NEdR calculated from the calibrated spectra of VBB, CBB, and ICT, respectively.

**Figure 12:**
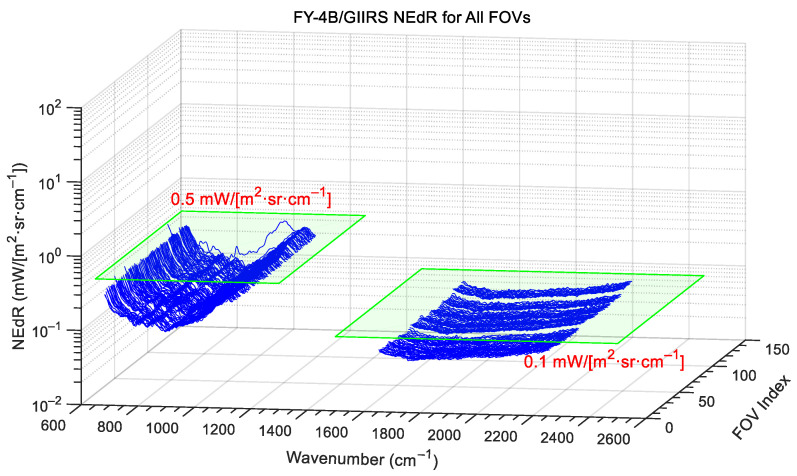
FY-4B/GIIRS NEdR spectra of all 128 FOVs in two bands. The zoomed inset shows that the NEdR of LWIR FOV-96 is an outlier with respect to the others, due to this pixel suffering from more noise.

As can be seen from Figure 11, the NEdRs from the three datasets are in a good agreement and are below the requirements of 0.5 mW/(m^2^·sr·cm^−1^) and 0.1 mW/(m^2^·sr·cm^−1^) for the LWIR and MWIR bands, respectively. Furthermore, the NEdRs for all 128 FOVs are shown in Figure 12. It can be seen that all LWIR FOVs, except for FOV-96, are below the requirement; and all MWIR FOVs are below the requirements and have a good agreement with each other. Actually, due to defects in the detector production, the pixels of LWIR FOV-16, -17, -31, and -96 suffer from more noise than others, and thus the NEdR spectra of these pixels are worse than others, especially for FOV-96.

### 4.3. Radiometric Calibration

#### 4.3.1. Radiance Modeling of Blackbody

The TVAC radiometric calibration of GIIRS-B aims to obtain the VBB radiance at different temperatures with a sufficient accuracy and stability. That is, in the dynamic range of the instrument measurement, no matter what the temperature of the VBB observed by GIIRS-B is, the calibrated radiance needs to meet the accuracy requirement of 0.7 K. However, inaccurate temperature measurements and inaccurate emissivity values of three blackbodies, short-term fluctuations in instrument temperature, the nonlinear response of the detector, and instrument noise will lead to inaccurate calibration radiance to varying degrees. For a non-ideal blackbody with its emissivity less than unity, the predicted radiance can be modeled by its temperature, emissivity, and reflected blackbody radiance from environment emissions. The following are the equations for the CBB, VBB, and ICT radiance modeling:

(6) $$\begin{array}{l} L_{CBB}\left(\sigma \right)=\epsilon_{CBB}B_{CBB}\left(T_{CBB},\sigma \right)+\left(1-\epsilon_{CBB}\right)B_{env}\left(T_{env},\sigma \right)\\ L_{VBB}\left(\sigma \right)=\epsilon_{VBB}B_{VBB}\left(T_{VBB},\sigma \right)+\left(1-\epsilon_{VBB}\right)B_{env}\left(T_{env},\sigma \right)\\ L_{ICT}\left(\sigma \right)=\epsilon_{ICT}B_{ICT}\left(T_{ICT},\sigma \right)+\left(1-\epsilon_{ICT}\right)B_{model}\left(T_{model},\sigma \right) \end{array}.$$

where *ε*_CBB_, *ε*_VBB_, and *ε*_ICT_ are the emissivities of the CBB, VBB, and ICT, respectively. *B*_CBB_, *B*_VBB_, and *B*_ICT_ are the ideal blackbody radiances with respect to the corresponding temperatures of *T*_CBB_, *T*_VBB_, and *T*_ICT_, respectively. *B*_env_ is the part of the radiance reflected from the blackbody that originates from the external environment when GIIRS views the CBB and VBB (with a corresponding temperature *T*_env_). *B*_model_ is the reflected radiance that originates from the self-emissions of the instrument’s internal components, such as the scanner baffle, scanning mirror, instrument frame, beamsplitter of the interferometer, and so on. This part requires reasonable modeling combined with ray trace to approximate it as an equivalent blackbody radiance with a temperature of *T*_model_.

For the blackbodies utilized in the TVAC test, the intrinsic uncertainties are tightly controlled by metrological calibration. The cavity emissivity (*ε*) of the blackbodies is generally greater than 0.99, and the physical temperature measurement uncertainty is maintained at approximately 0.05 K. While fluctuations in these baseline parameters, alongside random instrument noise, inherently contribute to the total radiometric uncertainty budget, their impacts are relatively stable and small (typically on the order of 0.1 K). In contrast, the uncorrected nonlinear response of the detector—particularly in the LWIR band—introduces an order-of-magnitude larger deviation (>1 K), making it the dominant source of radiometric calibration error. This outstanding nonlinearity issue is addressed in detail in the subsequent section.

#### 4.3.2. Nonlinearity Correction of LWIR Measurements

In the radiometric calibration of GIIRS-B, the induced calibration error caused by the nonlinear response of the LWIR detector is a significant contributor to measurement inaccuracy. Given that the LWIR module utilizes HgCdTe photoconductive detectors, the output electrical signals exhibit a non-proportional relationship with the input optical power, consistent with the inherent photoelectric conversion mechanisms of such devices [10]. Since the standard complex calibration model (Equation (4)) assumes that the instrument should be a linear response system, it is imperative to linearize the Fourier-transformed spectra prior to radiometric assignment.

The nonlinear response of an LWIR detector in math is usually represented by a polynomial fit between the output electrical signal and the input optical power. That is, the measured nonlinear (electronic) interferogram is fitted by a polynomial of the ideal linear (optical) interferogram. Since an interferogram signal consists of

(7) $$\begin{array}{l} I_{dc,m}=I_{dc,l}+\alpha \cdot I_{dc,l}^{2}+\beta \cdot I_{dc,l}^{3}+\cdots \\ I_{dc,m}=V_{m}+I_{ac,m}\\ I_{dc,l}=V_{l}+I_{ac,l} \end{array}.$$

where *I*_dc,m_ is the measured nonlinear interferogram with its direct current (DC) term *V*_m_ and alternating current (AC) term *I*_ac,m_, while *I*_dc,l_ is the ideal linear interferogram with its DC term *V*_l_ and AC term *I*_ac,l_. The higher-order harmonic terms of *I*_dc,l_ constitute the nonlinear signals. The symbols *α*, *β*, … represent the nonlinear fitting coefficients.

**Figure 13:**
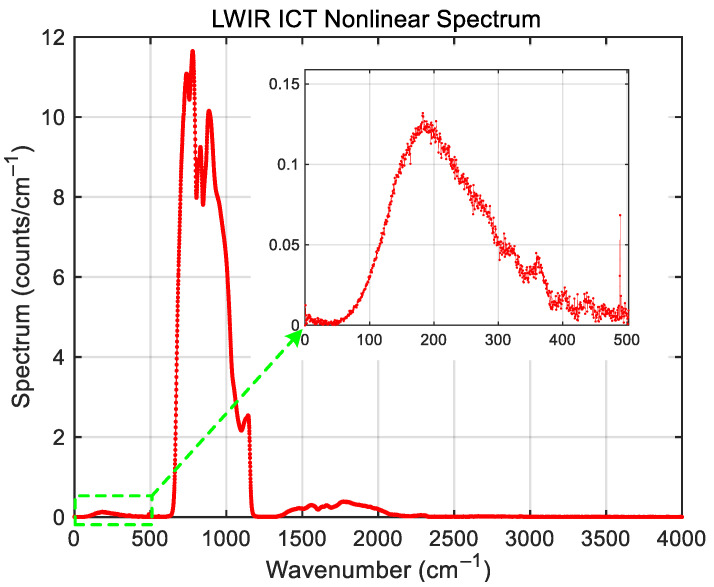
The nonlinear spectrum of ICT in the FY-4B/GIIRS LWIR band with the pseudo spectrum in the low wavenumber range shown in the zoomed inset.

The nonlinearity is a local function in the interferogram domain, especially in the region of centerburst. When transforming the interferogram into a spectrum, in addition to the resultant spectrum in the spectral region where the detector is sensitive (in-band), there are also artifacts that appear outside the detector-sensitive region (out-of-band) [11]. If the artifacts overlap in-band, that will distort the spectrum and thus degrade the radiometric calibration accuracy. An important and frequently occurring nonlinearity is a quadratic nonlinear response, which could be diagnosed and characterized from the presence of those artifacts. If the detection bandwidth of a detector is less than one octave, the large spectral artifact of the quadratic nonlinear response is located between zero and the long-wave cut-off, and the secondary spectral artifact is located beyond the short-wave cut-off of the detection band and can be described in terms of the autocorrelation with the true spectrum. The impact due to quadratic nonlinearity becomes more complex for measurements if the detection bandwidth is wider than one octave. In this case, the quadratic nonlinear response of the detector overlaps with the detection bandwidth and introduces additional offsets in the in-band spectrum. Figure 13 represents an example of an ICT LWIR nonlinear spectrum due to the quadratic nonlinearity. The in-band spectral distribution ranges from the long-wave cut-off wavenumber of 650 cm^−1^ to a short-wave cut-off wavenumber of 1150 cm^−1^, and the bandwidth is 500 cm^−1^. The first artifact covers the spectral region from 0 cm^−1^ to the long-wave cut-off wavenumber at about 500 cm^−1^, which is the same as the detection bandwidth. The second artifact starts at 1300 cm^−1^, which is twice the in-band starting wavenumber, and extends to the short-wave cut-off wavenumber at 2300 cm^−1^, with a bandwidth of 1000 cm^−1^, which is also twice the in-band of the first artifact bandwidth.

In addition, by performing polynomial expansion on Equation (7) and deriving the in-band spectrum expression, it can be found that the quadratic nonlinear response will also cause the overall in-band spectrum to be multiplied by a scaling factor, and it is this spectral scaling that produces the radiometric calibration deviation. Based on the above nonlinear spectral analysis, it can be inferred that the GIIRS-B LWIR interferogram is mainly affected by the quadratic nonlinear response, since there are no cubic and higher-order spectral artifacts in triple and higher-order frequency bands. Analogous analysis performed on the MWIR spectra revealed no discernable nonlinear artifacts and pseudo spectra like LWIR. In fact, the MWIR signal chain adopts a HgCdTe photovoltaic detector, which has a good linear responsivity according to the photoelectric conversion principle, and the circuit structure of a photovoltaic detector.

Referring to the methods used by similar instruments [12,13], a strategy of polynomial fitting to the measured interferogram is applied to correct the nonlinear interferograms into a linear interferogram, and the further Fourier-transformed spectrum is then also a linear spectrum. That is,

(8) $$V_{l}+I_{ac,l}=\left(V_{m}+I_{ac,m}\right)+a_{2}\cdot {\left(V_{m}+I_{ac,m}\right)}^{2}+{a_{3}\cdot \left(V_{m}+I_{ac,m}\right)}^{3}+\cdots .$$

This polynomial fitting strategy is deliberately selected over alternative methods like lookup tables or physical detector modeling due to specific hardware and system constraints. Lookup tables necessitate DC-coupled interferograms; however, the GIIRS-B amplifier circuit operates in an AC-coupled mode to maximize the spectral signal, inherently filtering out the required DC component. Furthermore, physical modeling is impractical because the observed nonlinearity is a complex, system-level effect encompassing both the detector’s inherent response and the subsequent electronic chain. Therefore, an iterative polynomial fitting approach—successfully proven on heritage instruments like CrIS and HIRAS—was adopted. This method effectively utilizes the theoretical radiance of the variable temperature blackbody (*L*_VBB_) as a reliable ground-truth reference to mathematically linearize the signal.

In addition, it is necessary to clarify the distinction between Equations (7) and (8). Specifically, Equation (7) describes the polynomial fitting of a nonlinear interferogram with an ideal linear interferogram, primarily to analyze the characteristics of the nonlinear spectrum. In contrast, Equation (8) describes the fitting of a linear interferogram with a nonlinear interferogram, with the primary purpose of correcting the nonlinear interferogram into a linear interferogram. In practice, for a correction procedure, the critical step is to find the polynomial fitting coefficients for nonlinear correction. As a rule of thumb, a second-order fitting polynomial is sufficient for nonlinearity correction, so the correction coefficient is the quadratic term coefficient of *a*_2_, with the first term set to 1 by default. Here, an iterative search method is used to find the optimal coefficient *a*_2_. This method is based on the fact that the instrument responsivity (InsResp) for a linear response system should maintain invariance even when observing scenarios with varying temperatures. Therefore, by determining an appropriate coefficient *a*_2_ through an iterative search to ensure convergence of the instrument responsivity dataset when observing the VBB at different temperatures, this coefficient can be further used to correct the nonlinear spectrum into a linear spectrum. The instrument spectral responsivity *R*_ins_ is deduced from the radiometric calibration equation and expressed as follows [14], which represents the ratio or gain of the instrument output spectrum (in digital units) with respect to the input radiance (in radiometric units):

(9) $$R_{ins}\left(\sigma \right)=\frac{\left|C_{VBB}\left(\sigma \right)\;-\;C_{CBB}\left(\sigma \right)\right|}{L_{VBB}\left(\sigma \right)}.$$

By performing a polynomial expansion on Equation (8), discarding the DC component, and calculating the Fourier transform of the AC component, the nonlinear corrected spectrum is expressed as follows [13]:

(10) $$\begin{array}{l} C_{l}\left(\sigma \right)=\left(1+2a_{2}V_{m}\right)\cdot C_{m}\left(\sigma \right)\\ C_{l}=FT\left\{I_{ac,l}\right\}\\ C_{m}=FT\left\{I_{ac,m}\right\} \end{array}.$$

Note that in addition to the nonlinear correction coefficient *a*_2_, the DC component *V*_m_ of the measured interferogram must also be included in the equation. However, in order to fully amplify the AC signal containing spectral information (i.e., *I*_ac,m_), the GIIRS-B detector amplifier circuit operates in AC coupled mode, and the DC term is filtered out and discarded by the onboard resistor–capacitor filter (RC filter). Therefore, the estimation method of the DC component involves first summing the raw spectrum along the wavenumber and then dividing the spectral integral by the interferometer modulation degree.

**Figure 14:**
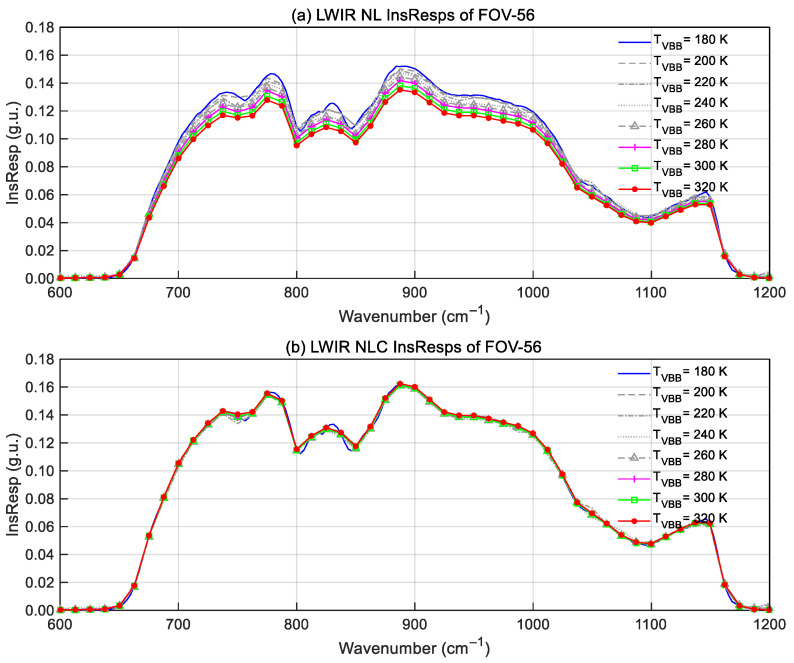
Comparison of GIIRS InsResps before and after the nonlinearity correction. (**a**) Responsivities of FOV-56 for different VBB temperatures before nonlinearity correction; (**b**) responsivities after nonlinearity correction.

Take FOV-56 as an example. Figure 14 shows the LWIR spectral responsivity of GIIRS-B calculated from TVAC VBB data at different temperatures. The VBB temperature increases from 180 K to 320 K at an interval of 20 K, and eight *R*_ins_(*σ*, *T*_VBB_) samples are calculated. Subplot (a) shows the InsResps before nonlinearity correction. By iteratively adjusting the coefficient *a*_2_, the InsResps are computed until the eight InsResps reach a convergence (subplot-b), with the corresponding *a*_2_ being the required nonlinear correction coefficient.

#### 4.3.3. Radiometric Calibration Accuracy Assessment

**Figure 15:**
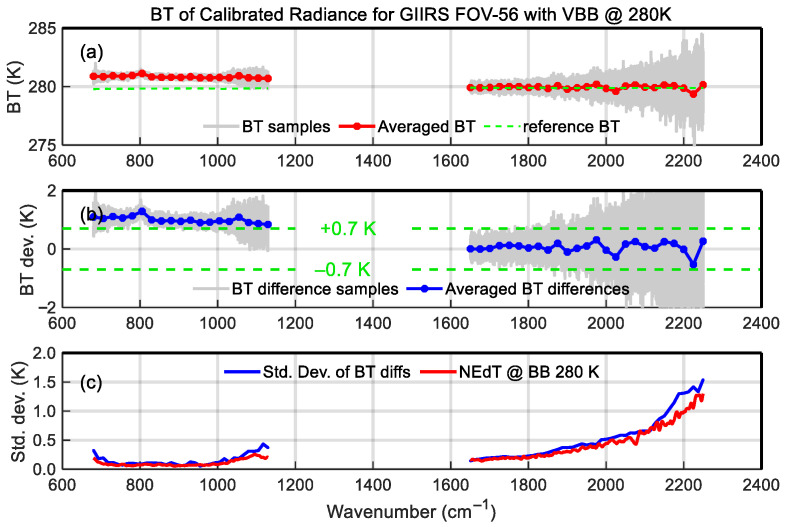
(**a**) The brightness temperature (BT) of FOV-56 calibrated radiance without nonlinearity correction with (**b**) BT difference and (**c**) standard deviations.

**Figure 16:**
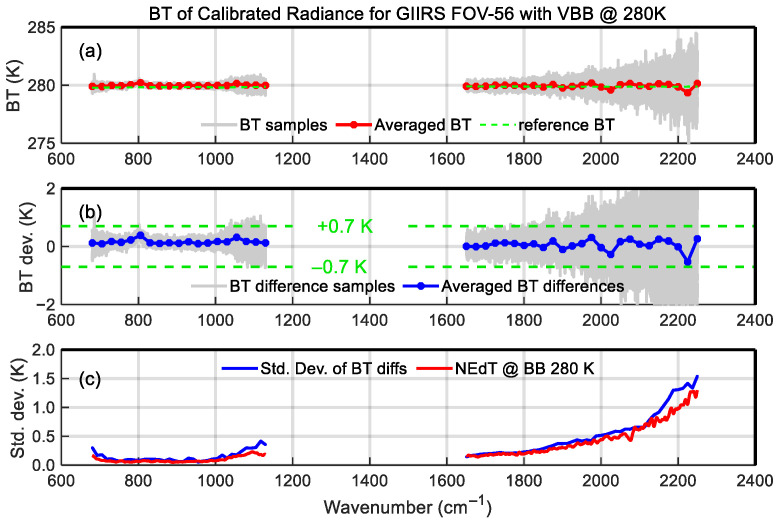
Same as Figure 15 except for the calibrated radiance with nonlinearity correction. (**a**) The brightness temperature (BT) of FOV-56 calibrated radiance with nonlinearity correction with (**b**) BT difference and (**c**) standard deviations.

The following is the comparison of the radiometric calibration accuracy before and after nonlinearity correction. Figure 15 shows the calibrated brightness temperature (BT) of 280 K VBB without nonlinear correction, with averaged bias and standard deviation of the samples. The top subplot in Figure 15 is the BT spectra, in which the fine solid gray lines represent the calibrated spectral samples, the blue line represents the sample averaged BT, and the green dashed line represents the reference spectrum calculated by the VBB radiance model. The middle subplot shows the calibration bias, in which the fine solid gray lines represent the bias samples, the blue line represents the mean of the bias samples, and the two dashed lines represent the upper and lower limits of the 0.7 K requirement of radiometric accuracy. The blue curve shown in the bottom subplot represents the standard deviation of the bias samples and is compared with the Noise Equivalent differential Temperature (NEdT) spectrum at the 280 K blackbody temperature calculated by NEdR. It can be seen from this figure that due to the nonlinearity-induced calibration error, the calibration deviation of LWIR BT has exceeded the requirement of 0.7 K and reached approximately 1 K; since MWIR calibration is not affected by nonlinearity, the calibration bias is within the requirement. Figure 16 shows the calibration results with nonlinearity correction. In this case, the LWIR calibration bias meets the requirement, and the biases of all channels are within the ±0.7 K limits.

As for the calibration results in the MWIR band, the averaged bias is within the 0.7 K requirement, but each calibrated spectral sample is affected by noise, especially in the range of 2000 to 2250 cm^−1^. Due to the presence of numerous water vapor absorption lines in the MWIR band, noise interference poses challenges for atmospheric spectral calibration and water vapor retrieval.

**Figure 17:**
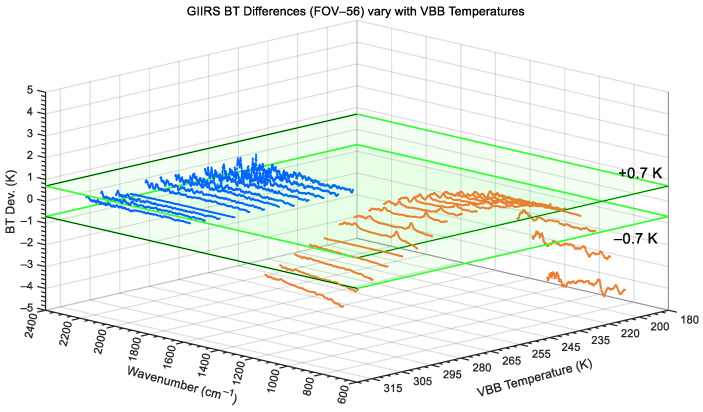
Without LWIR non-linearity correction, BT differences in GIIRS calibrated radiance at different VBB temperatures (taking FOV-56 as example).

**Figure 18:**
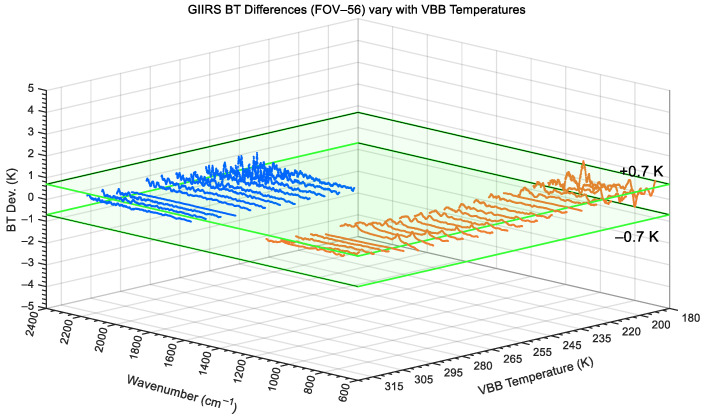
With LWIR non-linearity correction, BT differences in GIIRS calibrated radiance at different VBB temperatures (taking FOV-56 as example).

Taking FOV-56 as an example again, both Figure 17 and Figure 18 show the BT differences in the VBB calibrated radiances in the two bands for different temperatures from 180 K to 320 K. Figure 17 corresponds to the result of the long-wave infrared without nonlinear correction, while Figure 18 shows the calibration deviation with nonlinear correction. As the VBB temperature gradually increases, as shown in Figure 17, if the nonlinear correction is not applied, the BT differences in LWIR at low and high VBB temperatures can be as high as approximately 4.5 K. After nonlinear correction, as shown in Figure 18, the LWIR BT differences in the varying temperature range of 180 to 320 K are all within 0.7 K, although the lower the VBB temperature, the greater the noise interference.

In addition, it must be pointed out that when the VBB temperature is lower than 250 K, the MWIR spectrum will be severely contaminated by noise, and only the data measured at higher temperatures can be accurately calibrated. Therefore, in Figure 17 and Figure 18, the MWIR BT differences where the VBB temperature was lower than 250 K have been removed. In the preflight test, it is necessary to predict the in-orbit effective observation capability, that is, the dynamic range, of the GIIRS based on the VBB radiation calibration. In quantitative hyperspectral remote sensing, the effective observation dynamic range is strictly dictated by the instrument’s operational accuracy limits rather than its absolute detection limits, as inaccurate radiances actively degrade NWP data assimilation. Therefore, the TVAC test defines the valid dynamic range as the specific VBB temperature interval over which the calibrated brightness temperature (BT) deviation reliably remains within the required 0.7 K specification (as listed in Table 1). According to this criterion, the effective temperature range of the VBB is from 180 to 315 K in the LWIR band and is from 250 to 315 K in the MWIR band.

## 5. Conclusions

Preflight TVAC calibration is indispensable for establishing baseline calibration algorithms and predicting on-orbit performance. The instrument performance and calibration accuracy of the FY-4B/GIIRS directly impact the effectiveness of its data in regional weather forecasting and other applications. This study confirms that the FY-4B/GIIRS fulfills its design specifications across key metrics. With off-axis effect correction, the resulting ISRF conforms to the expected cardinal sine function with a spectral sampling interval of 0.625 cm^−1^. The spectral calibration accuracy is less than the 10 ppm requirement by comparing gas-cell-calibrated spectra with LBLRTM-simulated ones. The noise performance characterized by NEdR in the LWIR band is less than 0.5 mW/(m^2^·sr·cm^−1^) and, in the MWIR band, is less than 0.1 mW/(m^2^·sr·cm^−1^). Notably, the implementation of a nonlinearity correction method improved LWIR radiometric accuracy from >1.0 K to approximately 0.2 K, ensuring high-fidelity data (meeting the accuracy requirement of 0.7 K) across a reliable dynamic range of 180–315 K. While the MWIR band exhibits noise-limited performance at targets colder than 250 K, its accuracy remains compliant for typical meteorological scenarios in the well-calibrated range of 250–315 K. These preflight efforts provide the necessary characterization and coefficients to ensure the delivery of high-quality Level-1 radiance products, essential for advanced numerical weather prediction [15] and environmental monitoring [16].

## Figures and Tables

**Table 1:** FY-4B/GIIRS measurement requirements and accuracy specifications.

Parameter	Specification
Spectral Range	LWIR: 680~1130 cm^−1^ MWIR: 1650~2250 cm^−1^
Spectral Sampling	0.625 cm^−1^
NEdR	LWIR: <0.5 mW/(m^2^·sr·cm^−1^) MWIR: <0.1 mW/(m^2^·sr·cm^−1^)
Spectral Accuracy	<10 ppm
Radiometric Accuracy	<0.7 K
Spatial Sampling (@ s.s.p. ^1^)	12 km

^1^ sub-satellite point.

## Data Availability

The original contributions presented in this study are included in the article. Further inquiries can be directed to the corresponding author.

## Abbreviations

The following abbreviations are used in this manuscript:

FY-4B	FengYun-4B
GIIRS	Geostationary Interferometric Infrared Sounder
AIRS	Atmospheric Infrared Sounder
IASI	Infrared Atmospheric Sounding Interferometer
CrIS	Cross-track Infrared Sounder
HIRAS	Hyperspectral Infrared Atmospheric Sounder
NWP	Numerical Weather Prediction
TOA	Top-Of-Atmosphere
IR	Infrared
LWIR	Long-Wave Infrared
MWIR	Mid-Wave Infrared
Cal/Val	Calibration and Validation
TVAC	Thermal Vacuum
InsResp	Instrument Responsivity
ISRF	Instrument Spectral Response Function
BT	Brightness Temperature
NEdR	Noise Equivalent Differential Radiance
NEdT	Noise Equivalent Differential Temperature
FOR	Field Of Regard
FOV	Field Of View
Geo	Geostationary
PFM	Proto Flight Model
OPD	Optical Path Difference
IS	Integrating Sphere
LBLRTM	Line-By-Line Radiative Transfer Model
RMS	Root-Mean-Square Error
DS	Deep Space
ICT	Internal Calibration Target
CBB	Cold Blackbody
VBB	Temperature-Variable Blackbody
DC	Direct Current
AC	Alternative Current
CAS	Chinese Academy of Science
SITP	Shanghai Institute of Technical Physics
SISE	Shanghai Institute of Satellite Engineering

## References

[1] FY-4B Satellite. https://www.nsmc.org.cn/nsmc/en/satellite/FY4B.html.

[2] Smith W., Revercomb H.E., Woolf H., Huang H., Larar A., Zhou D., Kireev S., Tian J., Liu X. (2008). Ultra-spectral remote sounding: Background and future. Proc. SPIE.

[3] Hilton F., Armante R., August T., Barnet C., Bouchard A., Camy-Peyret C., Capelle V., Clarisse L., Clerbaux C., Coheur P.-F. (2012). Hyperspectral Earth Observation from IASI: Five Years of Accomplishments. Bull. Am. Meteor. Soc..

[4] Han Y., Revercomb H.E., Cromp M., Gu D., Johnson D., Mooney D., Scott D., Strow L., Bingham G., Borg L. (2013). Suomi NPP CrIS measurements, sensor data record algorithm, calibration and validation activities, and record data quality. J. Geophys. Res. Atmos..

[5] Qi C., Wu C., Hu X., Xu H., Lee L., Zhou F., Gu M., Yang T., Shao C., Yang Z. (2020). High Spectral Infrared Atmospheric Sounder (HIRAS): System Overview and On-Orbit Performance Assessment. IEEE Trans. Geosci. Remote Sens..

[6] Strow L.L., Motteler H., Tobin D., Revercomb H., Hannon S., Buijs H., Predina J., Suwinski L., Glumb R. (2013). Spectral calibration and validation of the Cross-track Infrared Sounder on the Suomi NPP satellite. J. Geophys. Res. Atmos..

[7] Revercomb H.E., Buijs H., Howell H.B., LaPorte D.D., Smith W.L., Sromovsky L.A. (1988). Radiometric calibration of IR Fourier transform spectrometers: Solution to a problem with the high-resolution interferometer sounder. Appl. Opt..

[8] Lee L., Qi C., Ding L. (2023). The instrumental responsivity effect to the calibrated radiances of infrared hyperspectral benchmark sounder. Proc. SPIE.

[9] Zavyalov V., Esplin M., Scott D., Esplin B., Bingham G., Hoffman E., Lietzke C., Predina J., Frain R., Suwinski L. (2013). Noise performance of the CrIS instrument. J. Geophys. Res. Atmos..

[10] Carli B., Palchetti L., Raspollini P. (1999). Effect of beam-splitter emission in Fourier-transform emission spectroscopy. Appl. Opt..

[11] Abrams M.C., Toon G.C., Schindler R.A. (1994). Practical example of the correction of Fourier-transform spectra for detector nonlinearity. Appl. Opt..

[12] Tobin D., Revercomb H.E., Knuteson R., Taylor J., Best F., Borg L., DeSlover D., Martin G., Buijs H., Esplin M. (2013). Suomi-NPP CrIS radiometric calibration uncertainty. J. Geophys. Res. Atmos..

[13] Wu C., Qi C., Hu X., Gu M., Yang T., Xu H., Lee L., Yang Z., Zhang P. (2020). FY-3D HIRAS Radiometric Calibration and Accuracy Assessment. IEEE Trans. Geosci. Remote Sens..

[14] Han Y., Chen Y. (2018). Calibration algorithm for cross-track infrared sounder full spectral resolution measurements. IEEE Trans. Geosci. Remote Sens..

[15] Kusano N., Burrows C. (2025). Impact Assessment of Chinese Hyperspectral Infrared Sounder in Preparation for MTG-S IRS. https://www.ecmwf.int/en/newsletter/183/news/impact-assessment-chinese-hyperspectral-infrared-sounder-preparation-mtg-s.

[16] Zeng Z., Lee L., Qi C. (2023). Diurnal carbon monoxide observed from a geostationary infrared hyperspectral sounder: First result from GIIRS on board Feng Yun-4B. Atmos. Meas. Tech..

